# A Three-Dimensional Stereotaxic MRI Brain Atlas of the Cichlid Fish *Oreochromis mossambicus*

**DOI:** 10.1371/journal.pone.0044086

**Published:** 2012-09-11

**Authors:** José M. Simões, Magda C. Teles, Rui F. Oliveira, Annemie Van der Linden, Marleen Verhoye

**Affiliations:** 1 Unidade de Investigação em Eco-Etologia, Instituto Superior de Psicologia Aplicada, Lisboa, Portugal; 2 Champalimaud Neuroscience Programme, Instituto Gulbenkian de Ciência, Oeiras, Portugal; 3 Bio-Imaging Lab, University of Antwerp, Antwerp, Belgium; National Institutes of Health/NICHD, United States of America

## Abstract

The African cichlid *Oreochromis mossambicus* (Mozambique tilapia) has been used as a model system in a wide range of behavioural and neurobiological studies. The increasing number of genetic tools available for this species, together with the emerging interest in its use for neurobiological studies, increased the need for an accurate hodological mapping of the tilapia brain to supplement the available histological data. The goal of our study was to elaborate a three-dimensional, high-resolution digital atlas using magnetic resonance imaging, supported by Nissl staining. Resulting images were viewed and analysed in all orientations (transverse, sagittal, and horizontal) and manually labelled to reveal structures in the olfactory bulb, telencephalon, diencephalon, optic tectum, and cerebellum. This high resolution tilapia brain atlas is expected to become a very useful tool for neuroscientists using this fish model and will certainly expand their use in future studies regarding the central nervous system.

## Introduction

Cichlid fish are one of the most successful taxa in vertebrate evolution. With over 3,000 species described so far, the family Cichlidae is the most species-rich family of vertebrates offering a scope of phenotypic and behavioral variation amenable to comparative analysis that makes them a popular model for evolutionary studies (e.g. [1]–[6]). Cichlid fish also present a wide variation, within closely related species, of their social behavior, ranging from territorial to shoaling species, and of their mating and parental care systems, including monogamous and polygamous breeding and paternal, biparental and maternal mouth-brooding or substrate-brooding species (e.g. [1], [6], [7]). The complexity and plasticity of their social behaviour are also remarkable (e.g. cooperative breeding, [8]; for a review of social plasticity in cichlid fish see [9] and of their cognitive abilities (e.g. transitive inference in the social domain, [10]), and recently, the impact of social complexity (i.e. dimension of social groups and existence of long-term relationships) on brain evolution in cichlids has been demonstrated [11]–[13]. Thus, cichlid fish offer a superb opportunity to study the neural and endocrine mechanisms underlying social plasticity and complexity and their evolution. In this regard, two African species have been mainly used in laboratory studies, the haplochromine *Astatotilapia burtoni* (e.g. [10], [14], [15]) and the tilapiine *Oreochromis mossambicus* (e.g. [9], [16]–[18]). This evo-mecho approach requires the identification and precise coordinates of relevant brain areas in a three-dimensional space, which would allow their precise measurement and manipulation (e.g. experimental lesions, micro-injections) for gain and loss of function studies. However, to the best of our knowledge, only partial 2D brain atlases based on histological sections are available for these species or for any other cichlid species [19]–[23].

In the last two decades the use of magnetic resonance imaging (MRI) to develop digital atlases was initiated with accurate human brain atlases (e.g. [24], [25]), but has been extended to non-human animals with a particular focus on mammals (e.g. mouse lemur, [26]; nemestrina monkey, [27]; mouse, [28]; rat, [29]; Rhesus macaque, [30]; marmoset monkey, [31]). The progressive technological developments of high-magnetic field MRI techniques also allowed imaging smaller animals, without losing resolution, such as the zebrafinch [32], the zebrafish [33], and the canary [34]. The three-dimensional and digital nature of MRI brain atlases offers more visualization and computational power when compared to classical 2D atlases. Although MRI atlases have a lower resolution than histological atlases they present numerous relevant advantages related with processing and analysis of relevant CNS structures: histological atlases use paraffin- or parlodion-embedded techniques which can cause tissue shrinkage during the dehydration and processing steps; after sectioning, the rehydration and staining methods are very hard to reproduce accurately from section to section; MRI-atlases are superior when analysing and measuring volumes of longer structures (like axon tracts and cranial nerves) due to its three dimensional nature, allowing a complete overview of the studied structure [35]. Thus, MRI neuroimage databases will have a crucial role in disseminating information about brain structure and function, not only in terms of the accurate description of species-specific brain features but also as a tool for comparative studies [36].

Here, we present the first three-dimensional stereotaxic atlas of the brain of a highly social cichlid fish (Mozambique tilapia, *Oreochromis mossambicus*) using MRI combined with a histological map as a guiding reference to label smaller brain nuclei, therefore relating the soft tissue contrast obtained with MRI with the cytoarchitectonic information provided by histology.

## Results

Here we present the first three-dimensional brain atlas for a cichlid fish species with complex social behaviour. The Mozambique tilapia 3D brain atlas is made available online at www.ispa.pt/ui/uie/ibbg/TilapiaBrainAtlas enabling the navigation through the whole brain.

MRI data are provided in raw, Amira and Analyse formats, which will allow users to fully browse and visualize the atlas as well as the delineations of brain nuclei using the commercial software Amira. It is also possible to visualize the MRI raw data, with limited ability, using free software, e.g. MRIcro. CT images of the skull and the skull delineation are also provided at the same location.

**Figure 1:**
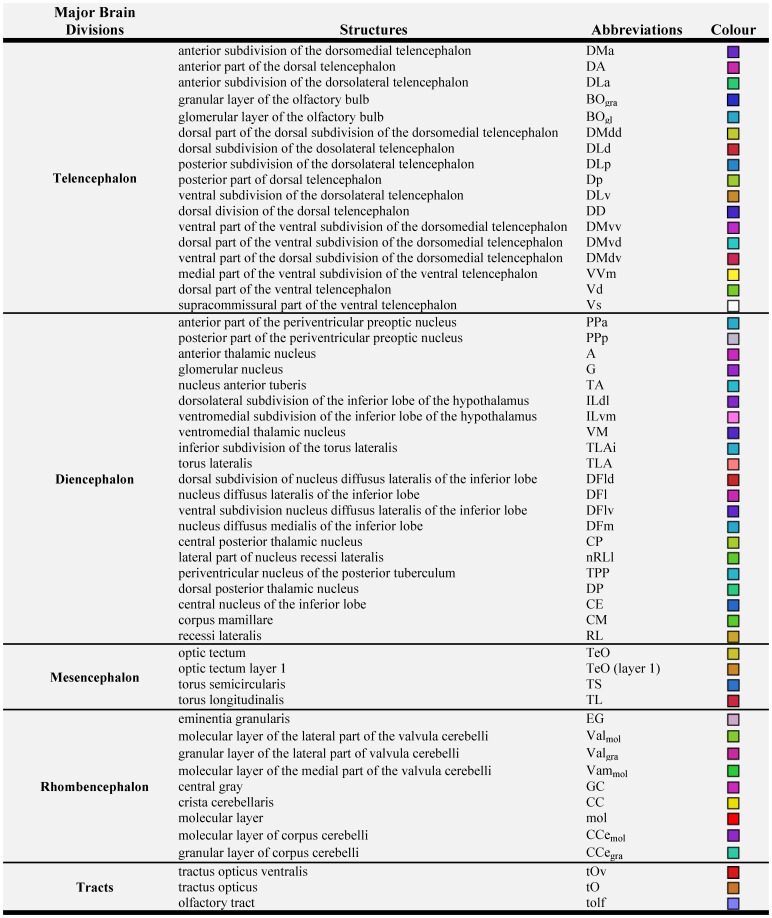
List of brain macroareas and tracts identified, as well as, all minor brain divisions, their abbreviation and chromatic identification on the 3D MRI reconstruction.

**Figure 2:**
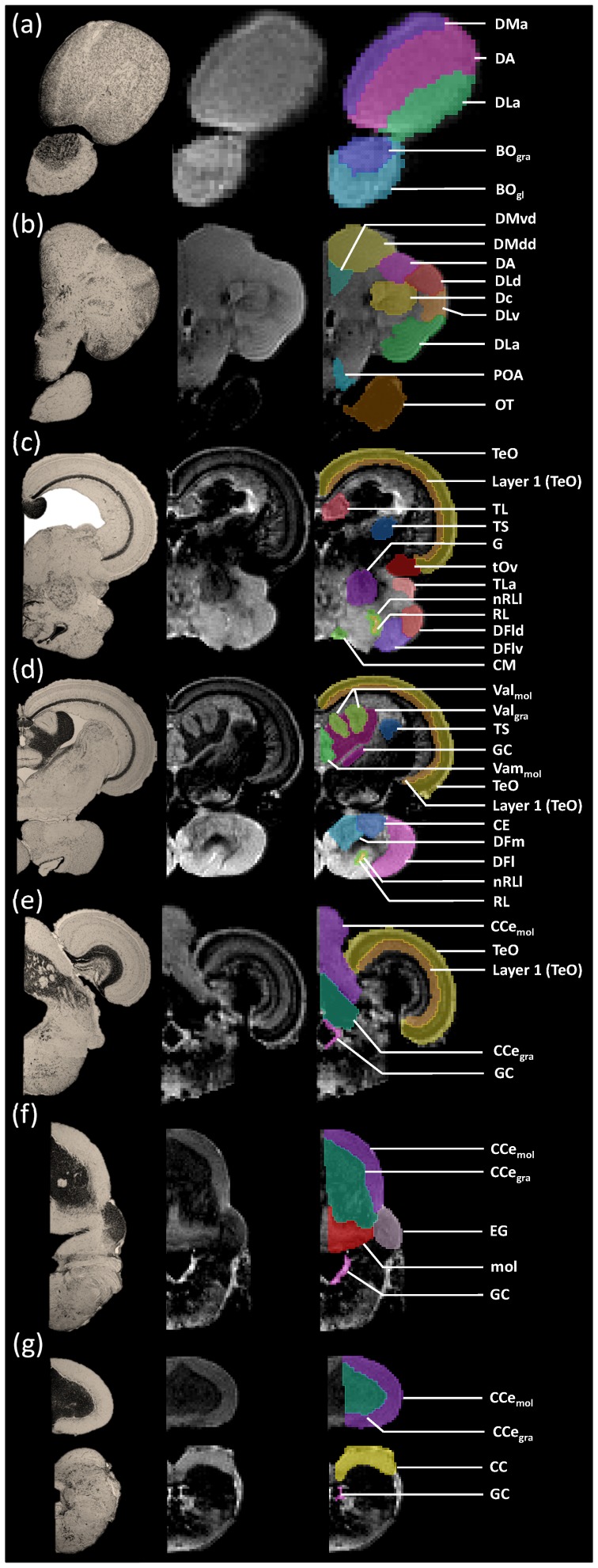
Comparison between Nissl stained histology sections (a) and MRI sections (b). On the left hand side is represented the olfactory bulbs and the beginning of the telencephalon. On the middle, we can see the end part of the optic tectum and diencephalon. Finally, on the right side is portrayed the cerebellum.

By using MRI in combination with classic histology, we developed a detailed three-dimensional atlas of the Mozambique tilapia brain, depicting several major and minor brain structures. Using T_2_-weighted and Nissl staining images in parallel for corresponding brain sections, a total of 54 brain structures (see Fig. 1) have been identified at an isotropic resolution of 50 µm. Our sequence and specimen preparation, which included Dotarem® as a paramagnetic contrast agent, enhanced the differentiation between regions in MRI images based on density, size and shape of neuronal cells. Thus, the depiction of nuclei in MRI images, is not much different from that using classic histology, since it is also possible to identify different tissue textures based on image contrast and pixel density pattern and position differences, to identify different cell agglomerations and nuclei. In contrast with classic paper histology atlases it is also possible to scroll readily between sections which provides critical insight when delimiting nuclei. Finally, with MRI one can label nuclei not only in a transverse perspective but simultaneously in all three dimensions. Nevertheless, the delineation of each nucleus was further supported by comparing MRI images to corresponding Nissl stained histological sections (Fig. 2). Therefore, all minor brain regions labelled on each MRI image, were subsequently rectified and confirmed using this comparative methodology. Although most structures are more conspicuous and detailed regarding cell morphology on the Nissl stained slides, they are nonetheless identifiable on the MRI images.

**Figure 3:**
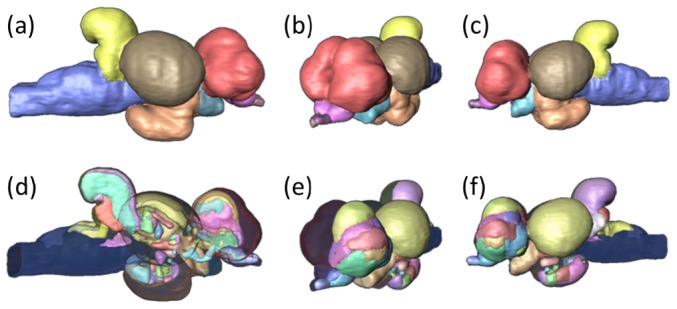
Rendering of the whole brain, depicting the major areas (a, b, c) as well as all the 54 delineated structures (d, e, f). Three different angles are presented to maximize the number of brain regions per image: (a), (d) right view; (b), (e) partial frontal view; (c), (f) left view. In the first row of images it is possible to define six major areas: telencephalon (red), olfactory bulbs (pink) and part of the olfactory tracts (purple), optic tectum (brown) and part of the optic tracts (light blue), diencephalon (orange), cerebellum (yellow) and the brain stem (blue). For a complete list of the small nuclei identified and the color code for the remaining images see Fig. 1.

Three-dimensional rendering of the delineated structures has been computed using Amira, and the rendering images of the whole brain depicting major brain divisions as well as the 54 delineated nuclei are provided in Fig. 3. These images provide a good approximation of the shape of each structure and allow an easy estimation of the relative volume of each nucleus (Fig. 1).

Using the intrinsic three-axis nature of MRI-based atlases, we established a stereotaxic coordinate system. The centre *x, y*, and *z* coordinates for each structure can be found in Table 1. As a zero point of the reference frame, we propose the intersection between the mid-sagittal and the mid-horizontal planes and the anterior commissure (AC). The latter, can be easily identifiable both on MRI and Nissl histology images, and the Y/Z (rostral/caudal and dorsal/ventral) axis passing through this point corresponds to the reference axis often used by electrophysiologists. Choosing an internal rather than an external landmark system was motivated by the fact that the shape of the fish's head may vary between sexes (males exhibit a concave dorsal head profile) and between adult and juvenile animals. Nonetheless, this approach will allow neurobiologists to accurately pinpoint different specific brain regions, when implanting cannulas or doing electrophysiology recordings. To facilitate these experimental methodologies we also imaged an entire tilapia head, where it is possible to visualize the relative position of the brain regarding its neighbouring structures (available online).

**Table 1:** List of smaller brain divisions organized by major areas and edifying their volume and *x*, *y* and *z* coordinates.

Major Brain Divisions	Structures	Volume (mm^3^)	Center X	Center Y	Center Z
**Telencephalon**	DMa	0,197	0,432	−1,522	0,419
	DA	0,579	1,323	−1,110	1,098
	DLa	1,143	1,539	−0,954	−0,165
	BO_gra_	0,248	0,480	−1,328	−0,543
	BO_gl_	0,507	0,501	−1,155	−0,824
	DMdd	2,223	0,762	−0,440	1,916
	DLd	0,793	2,001	−0,615	0,970
	DLp	0,614	1,876	0,274	0,301
	Dp	0,706	1,261	0,464	−0,017
	DLv	0,269	2,160	−0,766	0,448
	DD	0,360	1,502	0,298	1,391
	DMvv	0,809	0,256	−0,333	1,049
	DMvd	0,252	0,181	0,198	1,693
	DMdv	0,607	0,797	0,278	1,402
	VVm	0,040	0,103	−0,509	−0,107
	Vd	0,050	0,186	−0,582	0,439
	Vs	0,018	0,155	−0,122	0,206
**Diencephalon**	PPa	0,280	0,130	0,553	−0,598
	PPp	0,017	0,070	1,693	−0,677
	A	0,049	0,142	1,698	0,150
	G	0,264	0,915	2,508	−0,934
	TA	0,239	1,356	−0,167	0,901
	ILdl	0,092	1,096	2,108	−2,181
	ILvm	0,017	0,108	1,696	−0,330
	VM	0,030	1,844	1,901	−1,286
	TLAi	0,168	1,897	1,988	−0,879
	TLA	0,257	1,861	2,721	−1,610
	DFld	0,856	1,476	3,626	−1,660
	DFl	0,262	1,484	2,705	−2,111
	DFlv	0,573	0,335	3,621	−1,534
	DFm	0,053	0,304	1,981	−0,071
	CP	0,138	0,982	2,711	−1,821
	nRLl	0,029	0,118	1,919	−0,541
	TPP	0,029	0,179	1,988	0,230
	DP	0,405	0,899	3,596	−1,126
	CE	0,124	0,358	2,605	−1,614
	CM	0,240	1,356	−0,167	0,901
	RL	0,072	1,001	2,652	−1,810
**Mesencephalon**	TeO	5,418	1,676	2,554	1,134
	TeO (layer 1)	1,841	1,661	2,734	1,241
	TS	0,449	1,387	2,817	0,752
	TL	0,145	0,226	2,404	1,003
**Rombencephalon**	EG	0,537	1,223	4,815	0,877
	Val_mol_	0,230	0,566	3,252	1,368
	Val_gra_	0,479	0,617	3,128	1,009
	Vam_mol_	0,126	0,071	3,272	0,793
	GC	0,340	0,250	4,772	0,074
	CC	1,139	0,726	6,063	0,620
	mol	1,149	0,491	5,299	1,295
	CCe_mol_	2,605	0,533	4,977	2,467
	CCe_gra_	2,554	0,265	4,973	2,082
**Tracts**	tOv	0,433	1,660	2,062	−0,377
	tO	1,641	0,961	0,649	−0,933
	tolf	0,125	0,212	−2,111	−0,888

The coordinates of the structures were considered with respect to the origin at anterior commissure (in mm).

**Figure 4:**
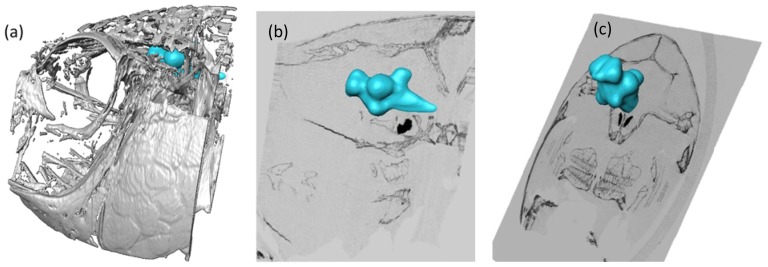
Overlap of MRI brain images (blue) with CT head data (light grey) in the Amira environment. (a) depicts a 3D reconstruction of the tilapia head based on the CT data set overlaid with a 3D tilapia brain. (b) and (c) show 2D sections of the head CT (sagittal and transverse views, respectively) and the tilapia's brain position in those perspectives.

We have also collected computerized tomography (CT) images that provide relevant information concerning the bony structure protecting and surrounding the brain. Using the Amira software, a three dimensional representation of this CT information has been registered with the MRI data set and a superimposed image of both data sets is illustrated in Fig. 4. This approach allows the integration of all collected information, which provides spatial coordinates regarding structures in the brain and around it.

## Discussion

Three-dimensional brain atlases have an enormous potential as gateways for navigating, accessing, and visualizing neuroscientific data [37]. An increasing number of recently published 3D MRI based brain atlases for emerging model organisms (e.g. zebrafinch [32], zebrafish [33] and canary [34]) highlight the advantages of using the MRI technique, despite their lower resolution when compared to classic histology and putative problems related with adjusting contrast and signal-to-noise ratio. These advantages are three-fold. First, digital MRI brain atlases, unlike classic histology sections, are not affected by shrinkage and physical distortions during sectioning and embedding of post-mortem brains. Thus, this technique provides a more precise way of processing neuroanatomical data, generating very precise stereotaxic coordinates, which can be used in electrophysiology and neuropharmacological studies. Second, and despite being limited by their resolution and contrast, MRI histology surpass the methodological constraints of classic histological sectioning techniques when analyzing complex structures [38]. It allows the morphological examination of anatomical brain structures in a three-dimensional space, the direct visualization of shapes and volumes of different brain structures, and a computerized sectioning of complex structures at arbitrary angles [32]. To ensure a rapid progress in this area, it will require increasing contribution of neuroinformatics, akin to the growing role of bioinformatics in other areas of biology. Finally, digital MRI atlases can be very useful tools to make generalizations about localization of various brain regions, their function and spatial structure at both the macroscopic and microscopic levels and to allow the comparison between different species.

In this paper we have managed to identify 54 brain nuclei in the brain of the Mozambique tilapia, which represents only roughly 30% of the brain areas that have been identified in the available 2D brain atlases for this species [21], [22]; where ca. 170 distinct structures have been described). The obvious reduction in the number of identifiable nuclei, due to the limitations in resolution characteristic of using the MRI technique, is surpassed by the neuroanatomical advantage of visualizing, in the same brain, volumes and shapes of different nuclei in a three dimensional space and to be able to determine their location based on a more precise coordinate system. Consequently, this provides a powerful tool for neuroscientists to better calculate the ideal orientation of the brain for electrophysiological recordings, stereotactic injections or brain sectioning [32]. The combined use of histological and MRI images allows a better understanding of the spatial relationships of different brain structures by linking the resolution provided by the cytoarchitectural detail of classic histology, with the 3-D representations provided by the MRI technique (e.g. [31], [34]).

A comparison between our 3D MRI atlas to that of zebrafish [33] shows that here we can distinguish a larger number of telencephalic and diencephalic nuclei but a lower number of the smaller nuclei located in more caudal areas (e.g. rhombencephalon, brain stem). These structures are clearly identifiable in the histological sections, but very hard to delimitate precisely in our MRI sections. This is due to the fact that we have used a less powerful MRI scanner than the one used for zebrafish (i.e. a 9.4 T that allowed an isotropic resolution of 50 µm in tilapia vs. a 16.4 T that allowed an isotropic resolution of 10 µm in zebrafish). Thus, the availability of more potent MRI scanners in the near future will play a pivotal role in the development of higher resolution 3D brain atlases for small model organisms.

Although cichlid species are excellent models for comparative social neuroscience studies, given the complexity and diversity of their social systems described above, the data published so far has used very gross neuroanatomical measures [11]–[13] and detailed neuroanatomical data is currently only partially available for two species [telencephalon and diencephalon of *Astatotilapia burtoni*: 19, 23; and whole brain of *O. mossambicus*: 21 and this paper]. A comparison of forebrain of these two species shows a very similar organization that is typical of percomorphs. The dorsal telencephalon of both species is divided into three highly elaborated (i.e. with many identifiable cell groups) areas, dorsolateral (Dl), dorsomedial (Dm) and dorsocentral (Dc), and two more uniform dorsal (Dd) and posterior areas (Dp). The subdivisions within each of these areas do not always match between the two species but at present it is difficult to understand to what extent these differences in nomenclature reflect real cytoarchitectural differences or different interpretations among authors. Future studies using genetic markers may help to solve these divergences. Two cell groups are clearly identified in both cichlid species that have not been described before in other teleost species: a granular zone in Dld (named Dl-g in *A. burtoni*) and Dcm (named Dm-2 in *A. burtoni*) (see sections 3/24 to 5/24 on the accompanying website to this paper). Once more, future studies are needed to establish the function of these cell groups that may represent specializations of the cichlid telencephalon. At the level of the ventral telencephalon the main cell groups described for other teleosts were also found in both species: ventral (Vv), dorsal (Vd) and supracommissural (Vs) nucleus. The diencephalon is also highly conserved in both species, with minor differences between the two species. In the hypothalamus, the diffuse nucleus of the inferior lobe in tilapia is preceded by the dorsolateral subdivision of the inferior lobe (ILdl), which will further subdivide in the dorsal and ventral subdivision of nucleus difusus lateralis of the inferior lobe, DFld and DFlv respectively. In contrast, in *A. burtoni* the diffuse nucleus of the inferior lobe (Dn) is located anatomically at the same positions of ILdl and no further divisions occur [19]. Also in the posterior tuberculum, the mammillary body lies ventrally to the preglomerular commissural nucleus (PGCn) in *A. burtoni* whereas in tilapia this structure is located ventral to the Nucleus of the posterior tuberculum (TP). In conclusion, although the three-dimensional brain atlas of tilapia presented here cannot be used accurately with other cichlid species, it offers a detailed description of a cichlid brain which, given the similarities described above between the two cichlid species studied so far, can be used with caution as a reference guide for investigators starting to work in other cichlid models.

In summary, the high resolution 3D brain atlas presented here is expected to become a very useful tool for neuroscientists already using tilapia as a model organism and will contribute to make this species more usable in future studies of the central nervous system. As a first step in this direction we have created a free access website for the tilapia 3D brain atlas and we are developing the tools that will allow the annotation by authorized visitors of the available online brain atlas with multiple information (e.g. distribution of different receptors, neurotransmitters and neuropeptides; gene expression patterns; adult cell proliferation areas and newborn cell migration routes; etc.).

## Materials and Methods

### Specimen Preparation

To collect MRI images, two males and two females (standard length: 10.7±1.8 mm) were perfused transcardially, first with a phosphate-buffered saline solution (PB 0.2 M), to clear the vasculature, followed by a solution of Paraformaldehyde (2%) in Dotarem® (1%), to fix the tissue with a paramagnetic MR contrast agent. The fish were postfixed in a mixture of PFA/Dotarem for 5 days. The day before imaging, the brains of three fish were removed from the skull and transferred to a polypropylene tube filled with Fluorinert®, a proton-free susceptibility-matching fluid and scanned with the highest resolution to enable a further identification of brain nuclei (Brain Imaging). The other perfused fish (N = 1 adult male) was scanned to stereologically study the brain's position inside the head and skull (Head Imaging). Although three data sets were registered to create a model tilapia brain unfortunately, due to technical issues, the quality of the registration was limited in comparison to individual data sets and therefore, we have used a single dataset from an adult male. However, it should be stressed that the three scanned brains were visually compared, to ascertain the representativity of the data set shown, and no differences were observed.

This study was performed in strict accordance with the recommendations of the Direcção Geral de Veterinária, the Portuguese National Authority for Animal Health, and the protocol was approved by their ethics committee (Permit Number: 0420/000/000/2007). All surgery was performed under MS222 anesthesia, and every effort was made to minimize suffering.

### Histological data

For the histology, four adult tilapia (2 males and 2 females; standard length: 9.6±1.1 mm) were perfused using a similar protocol to the one described above but without the MR contrast agent. After perfusion, the brains were removed from the skull, post-fixed for 1 h in PFA (2%) and transferred to a formalin solution (10% buffer). After fixation, brains were dehydrated (Leica TP1020) and embedded in paraffin before they were cut in transverse sections at 10 µm and mounted serially on glass slides. The sections were then deparaffinised for 10 min at 70°C, rehydrated and stained with a Nissl staining protocol. Finally, the sections were dehydrated and coverslipped with DPX mounting medium (Merck). Since there were no obvious sex differences in brain anatomy the histology figures used here represent the brain of an adult male, which is consistent with all other figures shown.

### MR image acquisition

**Brain Imaging**: MRI scanning was performed on a 9.4 T horizontal bore Magnetic Resonance Imaging system (Bruker BioSpin MRI GmbH, Ettlingen, Germany) using the standard Bruker cross coil setup, being a quadrature transmit volume coil (inner diameter 72 mm) and a quadrature receive surface coil, designed for mice brain. Horizontal images of the Tilapia brain were acquired using a fat-suppressed T_2_-weighted three-dimensional RARE sequence with the following parameters: acquisition bandwidth of 33 kHz, TE/TR = 30/350 ms, echo train length = 2, 8 averages, a field of view of (13.5×8×10) mm^3^ and an acquisition matrix of (270×160×200), resulting in a nominal spatial resolution of (50×50×50)µm^3^. The total acquisition time was 12.6 h.

**Head Imaging**: Images were acquired using the same MRI equipment, using the same quadrature volume coil both for transmission and receiving. For the whole head imaging was used a fat-suppressed T_2_-weighted three-dimensional RARE sequence with the following parameters: acquisition bandwidth of 50 kHz, TE/TR = 26/350 ms, echo train length = 2, 4 averages, a field of view of (80×40×30) mm^3^ and an acquisition matrix of (400×200×150), resulting in a nominal spatial resolution of (200×200×200)µm^3^. The total acquisition time was 5.8 h.

### CT acquisition

In order to acquire images of the skull, the whole head of a perfused adult male was also scanned with an X-ray micro-CT system (Skyscan 1076, Belgium, focal spot size of 5 µm, energy range of 20–100 keV). An image data with matrix (1649×2448×372) and resolution of (18×18×18) µm^3^ was achieved.

### Image post-processing

Brain and nuclei delineation was done manually using Amira software (Mercury Computers Systems, USA). Segmentation was done slice-by-slice in a transverse perspective and posteriorly confirmed systematically in the two other orthogonal views (axial and sagittal). Major brain subdivisions (Telencephalon, Diencephalon, Mesencephalon, Rhombencephalon) were first delineated, followed by structures which presented more distinct boundaries (e.g. olfactory bulbs, optic tectum and corpus cerebellis), which helped identifying smaller nuclei. In addition, histology sections were used as reference for the location and boundaries of smaller structures. Histology sections were digitised, juxtaposed to MRI images and together analysed in order to more precisely delineate all nuclei. Nuclei which did not present clear contrast differences/boundaries in the MRI were not considered, despite being histologically identifiable.

Nuclei volume measurements were calculated using the Material Statistics function in the Amira software. Uploading the MRI and nuclei delimitation data with the free software MRIcro, using the same procedures described by Poirier et al. [32], allowed to extract the stereotaxic coordinates for each nuclei.

Co-registration of CT images to the MRI brain atlas was performed with Amira, by an affine transformation of the CT data – down-sampled to (70×70×70) µm^3^ – to the MRI.

### Neuroanatomical analysis

There is a rich tradition in comparative neuroanatomy of fish that has prompted the emergence of different nomenclatures for brain structures of ray-finned fishes (e.g. [39]–[44]. In this paper we adopted the nomenclature used by [21] in the previously published 2D brain atlas of this species. This nomenclature follows the scheme proposed by [42] and [43], but introduces new terms that reflect some peculiarities of the cichlid brain.
